# Genome Sequence of *Salipaludibacillus* sp. Strain CUR1, Isolated from an Alkaline-Saline Lake in Rajasthan, India

**DOI:** 10.1128/mra.01092-21

**Published:** 2022-06-06

**Authors:** Dhankesh Meena, Janmejay Pandey, Rajesh Vasita, Shiv Swaroop

**Affiliations:** a Department of Biochemistry, School of Life Sciences, Central University of Rajasthan, Ajmer, Rajasthan, India; b Department of Biotechnology, School of Life Sciences, Central University of Rajasthan, Ajmer, Rajasthan, India; c School of Life Sciences, Central University of Gujarat, Gandhinagar, Gujarat, India; University of Maryland School of Medicine

## Abstract

We report the complete genome sequence of *Salipaludibacillus* sp. strain CUR1, which was isolated from Sambhar Lake (a soda lake) in Rajasthan, India. The whole-genome sequencing of this strain has been done to explore the industrially important hydrolytic and extracellular enzymes that can be active under high-salt and high-pH conditions.

## ANNOUNCEMENT

A rod-shaped, Gram-positive, nonmotile, and endospore-forming bacterium that is a member of the *Bacillaceae* family in the order *Bacillales*, class *Bacilli* (1–3), is reported here. *Salipaludibacillus* is known to produce several industrially important extracellular enzymes (4). Soil sediment collected from Sambhar Lake in Rajasthan, India, was serially diluted and plated on Luria-Bertani (LB) agar containing 7.0% (wt/vol) NaCl (pH 9.0) at 37°C to obtain morphologically distinct single colonies. A single colony was inoculated and grown in LB broth containing 7.0% NaCl (pH 9.0) at 37°C for 3 days. Genomic DNA was extracted by the phenol-chloroform method (5). The purified DNA was checked by agarose gel electrophoresis and a NanoDrop spectrophotometer.

The preparation of a paired-end (PE) sequencing library was performed using a TruSeq Nano DNA library preparation kit (6). The concentration of the library was 1,879 pg/μL, with an average size of 428 bp. The PE library was sequenced on an Illumina platform with 2 × 150-bp chemistry to generate ~1.7 Gb of data/sample, with a total of 11,350,378 reads. High-quality clean reads were obtained after processing of the sequenced raw data by removing adaptor sequences, ambiguous reads, and low-quality sequences using Trimmomatic v0.36 (7). *De novo* assembly of high-quality PE reads for the sample was performed using Velvet v1.2.10, and the assembly was optimized with a k-mer value of 121 (8). The scaffolds were further gap filled by GapCloser v1.12 software using PE read information (9). The size of the genome was 4,396,493 bp in 96 contigs (*N*_50_, 154,400 bp; *L*_50_, 9), with a GC content of 42.44% and coverage of 375×. Gene identification was performed by the NCBI Prokaryotic Genome Annotation Pipeline (PGAP) v5.3 (10). A total of 4,259 genes were identified in the genome, of which 4,129 were protein coding. The genome contains 19 rRNA genes and 76 tRNA genes. When protein-coding genes were searched for similarity against the NCBI nonredundant, UniProt, Pfam, and Clusters of Orthologous Genes (COG) databases using BLASTp v2.8.1+ (https://blast.ncbi.nlm.nih.gov/Blast.cgi?CMD=Web&PAGE_TYPE=BlastNews) with an E value threshold of 1*e*−05, hits were obtained for 4,089, 3,207, 2,676, and 3,311 genes, respectively. Function annotation of nonredundant annotated genes using Blast2GO CLI v1.4.1 (11) revealed that 1,806 genes were related to various biological processes, 1,348 genes were involved in the formation of cellular components, and 1,985 genes were associated with molecular functions. The genome was found to be 99.34% complete, as estimated with CheckM v1.1.3 (12). Default parameters were used for all software unless otherwise specified.

The genome sequence of strain CUR1 will shed light on the survival strategy of this haloalkalotolerant organism and its production of extracellular hydrolytic enzymes. We also intend to study the genes involved in the production of various advance bacterial biopolymers.

### Data availability.

This whole-genome shotgun project has been deposited in DDBJ/ENA/GenBank under the accession number JAIWPE000000000. The version described in this paper is version JAIWPE010000000. The raw data are available under SRA accession number SRX13075965. The BioSample accession number is SAMN18347004.
